# Explaining the COVID19 mortality growth rate: an empirical analysis of Israeli cities

**DOI:** 10.3389/fpubh.2025.1548294

**Published:** 2025-07-09

**Authors:** Yuval Arbel, Yifat Arbel, Amichai Kerner, Miryam Kerner

**Affiliations:** ^1^Sir Harry Solomon School of Economics and Management, Western Galilee College, Acre, Israel; ^2^Department of Mathematics, Bar Ilan University, Ramat Gan, Israel; ^3^School of Social Sciences, Banking and Finance Program, Bar Ilan University, Ramat Gan, Israel; ^4^The Ruth and Bruce Rappaport Faculty of Medicine, Technion, Israel Institute of Technology, Haifa, Israel; ^5^Department of Dermatology, HaEmek Medical Center, Afula, Israel

**Keywords:** COVID-19, mortality, population size, median age, socio-economic ranking

## Abstract

**Background:**

The severe acute respiratory syndrome coronavirus 2 (SARS-CoV-2) is an infectious virus, which has generated a global pandemic. Since December 20, 2020, Israel was one of the first countries to vaccinate its population. This study analyzes the weight of four covariates on a daily mortality growth rate from SARS-COV2 virus. These include population size, median age, a socio-economic ranking at a city level, a date variable and a dummy variable that equals 1 for post-vaccination and 0 for pre-vaccination era.

**Method:**

Regression analysis, where each variable is converted to the standard normal distribution function. This methodology permits the estimation of variations in daily mortality growth rates, where all the covariates are given in comparable units of measurement (one standard deviation). Consequently, the coefficients of this regression have to be measured as absolute value weights.

**Results:**

Findings suggest a rise in projected mortality growth rate with population-size and median age, and a drop with socio-economic ranking and vaccination availability. Of the four investigated covariates, population size and median age of the city have the highest weight, whereas socio-economic ranking and vaccination availability have the lowest weight.

**Conclusions:**

In an effort to reduce the mortality of severe coronavirus disease (COVID19) patients, greater priority should be given to larger cities with a relatively older population profile. In particular, policies should strive for better coordination at a municipal level between health and municipal and welfare services, particularly in large cities.

## 1 Introduction

COVID-19 is regarded as the second most consequential epidemic in modern history, following the Spanish flu of 1918–1921, largely due to its extensive global reach and mortality (1–3). A central concern throughout the pandemic has been the scale of global fatalities. By early October 2021, COVID-19 had resulted in 4.8 million deaths worldwide. While this number is considerably lower than the estimated 35 million deaths caused by the Spanish flu, the comparison becomes much more striking when adjusted for today's global population. If the Spanish flu were to occur under current demographic conditions, it could have caused as many as 150 million deaths (3). Additionally, forecasts indicate that the total number of deaths from COVID-19 could continue to rise significantly in the coming years (4).

Despite these global comparisons, the impact of COVID-19 has varied greatly from one country to another. In the United States, the toll has been especially heavy. As of October 7, 2021, the country had recorded 710,173 COVID-19 deaths—exceeding the estimated 675,000 American fatalities from the 1918–1919 Spanish flu (5). This milestone highlights the extraordinary burden the current pandemic has placed on the U.S., despite a century of advancements in medical science and public health systems.

In contrast, Israel's experience has been markedly different. Although it faced many of the same global challenges, its official COVID-19 death toll has remained comparatively low. This contrast highlights the uneven distribution of the pandemic's effects and positions Israel as a noteworthy case in the broader international response (4).

Israel provides a notable case study during the COVID-19 pandemic, featuring:

1. Uneven distribution of population density, and high levels of urbanization which might exacerbate the spread of the virus (6, 7).
2. Variations in household income and socioeconomic status (8).
3. More than 50% of the population being fully vaccinated by March 2021 following an early nationwide vaccination campaign (9).

This study aims to investigate how vaccination rates, city population size, socioeconomic status, and median resident age in 167 local authorities affect the daily COVID-19 mortality growth rate. It utilized panel data tracking mortality rates from March 11, 2020, to September 21, 2021, with vaccines becoming available from December 20, 2020.

In contrast to previous studies that used diverse measurement units, this research applied normalized variables in the regression model. The findings show that among the four factors examined, city population size and median age have the most significant impact, whereas socioeconomic status and vaccination availability have the least. The size of the city population is the most influential factor on expected mortality rates due to increased interactions in densely populated areas. Additionally, older age groups are at a higher risk of infection and mortality (2, 10, 11). Conversely, socioeconomic status and the initiation of vaccination campaigns have a negative effect on mortality rates.

The article is structured as follows. Section 2 describes the descriptive statistics. Section 3 presents the methodology and Section 4 presents the results. Finally, Section 5 concludes and summarizes.

## 2 Descriptive statistics

This section describes the variables used in the empirical model. The descriptive statistics of the variables are presented in Table 1. These data are official figures provided by the Israeli Ministry of Health and are presented here as they appear in the original database.

**Table 1:** Descriptive statistics.

**Variable**	**Description**	**Obs**	**Mean**	**SD**	**Min**	**Max**
*t*	Date variable from 11 (= March 11, 2020; the first documentation of COVID19 cases) to 560 (= September 21, 2021)	70,085	341.40	134.96	11	560
Δ*ln*(Cum_Deaths) = ln(Cum_Deaths)_*t*_−*ln*(Cum_Deaths)_*t*−1_	Approximated daily mortality growth rate from SARS-COV2 virus in each of the 167 Israeli municipalities where Cum_Deaths is the accumulated number of deaths	70,085	0.0013	0.007	0	0.2113
*Dum_vaccine*	1 = post-vaccine era (December 20, 2020-September 21, 2021); 0 = pre-vaccine era (March 21, 2020–December 19, 2020)	70,085	0.6507	0.4767	0	1
*MedianAge*	Median age of city inhabitants in years	70,085	28.75	6.40	11.44	40.73
*PopulationSize*	Urban population size	70,085	52,172.13	96,486.48	5,446	865,721
*RANK*2013	Socio-economic ranking on a scale of 1 = the lowest to 255 = the highest	70,085	118.1978	74.1146	2	253

The dataset Refers to 167 Israeli municipalities (*i* = 1, 2, 3, ⋯ , 167) and up to 550 days (*t* = 11, 12, 13, ⋯ , 560), where *i*×*t* = 70, 085. Note that the panel is unbalanced. For many localities, data is missing for numerous days. Additionally, in small and isolated localities where mortality rates are low, the number of deaths often remains unchanged.

The study utilizes data from 167 local authorities in Israel (*i* = 1, 2, 3, ⋯ , 167) over a period of up to 550 days (*t* = 11, 12, 13, ⋯ , 560), resulting in a total of *i*×*t* = 70, 085 observations.^1^ The number of days is calculated as (550 = 560 − 11+1). The time variable *t* corresponds to calendar dates ranging from day 11 (March 11, 2020, marking the first recorded COVID-19 cases in Israel) to day 560 (September 21, 2021). Throughout the article, the indices *i* and *t* are used or omitted as appropriate, depending on the context.

It is also important to note that the panel used in this study is unbalanced; otherwise, we would expect 167 × 550 = 91, 850 observations. However, for many localities, data are missing for a substantial number of days. Moreover, in small and isolated localities with low mortality rates, the number of recorded deaths often remains unchanged over time.

In each of the 167 cities and towns in Israel, the dependent variable *ln*(Cum_Deaths) equals ln(Cum_Deaths)_*t*_−*ln*(Cum_Deaths)_*t*−1_ and reflects the estimated daily mortality rate from the SARS-COV2 virus (*Cum_Deaths* is the accumulated number of deaths). Johnston and Dinardo [(12), p. 42–45] demonstrate that logarithmic differences between two adjacent data of a time series reflect an approximation of a constant rate of percentage change. Thus, ln(Cum_Deaths)_*t*_−*ln*(Cum_Deaths)_*t*−1_reflects the daily change in the mortality rate.^2^ It is worth noting that the difference between the cumulative values on two consecutive days corresponds to the number of deaths recorded on the latter day.

According to Table 1, and with respect to the dependent variable *ln*(Cum_Deaths), the average daily mortality rate is 0.13% and the standard deviation is 0.7%. The maximum daily increase rate in mortality is 21.13%. This figure was obtained in Jerusalem from 17 deaths on April 9, 2020, to 21 deaths on April 10, 2020 [an exact calculation yields an increase of $\frac{21}{17}-1\;=\;$23.52%, and an approximated calculation *ln*(21)−*ln*(17) = 21.13%].

The independent variables include *Dum_vaccine*; *median_age*; *Population size* and *RANK2013. Dum_vaccine* equals 1 in the post-vaccination era (December 20, 2020–September 21, 2021); 0 = pre-vaccination era (March 21, 2020–December 19, 2020). According to Table 1, during the sample period, the COVID19 vaccine was available, and the population was vaccinated 65% of the time. With regard to *MedianAge* (*median age* of city residents in years), the median average age is 28.75 years and the standard deviation is 6.40 years. The median age variable was calculated by the Israeli Ministry of Health and the Central Bureau of Statistics, which determined the median age in each locality based on the ages of all residents. The median age ranged from 11.44 to 40.73 years. With regard *to population size* (urban population), the average is 52,172 inhabitants and the standard deviation is 96,486 inhabitants. The minimum is 5,446 persons (Kfar Vradim in the Western Galilee) and the maximum is 865,721 persons (Jerusalem). With regard *to RANK2013* (socioeconomic ranking of the authorities on a scale of 1 = lowest to 255 = highest), the average is 118 and the standard deviation is 74. The minimum and maximum rank (2, Shaqib al-Salam) and 253, Lehavim) are located in the Negev.

## 3 Methodology

Consider the following models:


(1) $$\begin{array}{l} \Delta ln{\left(Cum\_Deaths\right)}_{i,t}\;=\;\alpha_{0}+\alpha_{1}Dum\_Vaccine_{i,t}\times t\\ \;+\alpha_{2}MedianAge_{i}+\alpha_{3}PopulationSize_{i}\\ \;+\alpha_{4}RANK2013_{i}+\mu_{i,t} \end{array}$$

(2) $$\begin{array}{ccc} \mu_{1i,t}\; & = & \;a_{1i}+\in_{1,i,,t} \end{array}$$

where the municipalities index is denoted by *i* (*i* = 1, 2, 3, ⋯ , 167), the variable *t* is the date index from 11 (= 11 March 2020; first record of cases COVID19) to 560 (= 21 September 2021); Δ*ln*(Cum_Deaths)_*i, t*_ is the approximated daily SARS-COV2 mortality decline or growth rate. The independent variables are: Dum_Vaccine_*i, t*_×*t*; MedianAg*e*_*i*_; PopulationSiz*e*_*i*_ and RANK2013_*i*_. Except Dum_Vaccine_*i, t*_×*t*, all independent variables are generic and constant over time in the same municipality. Finally, ∈_1, *i*,, *t*_ is the random disturbance term satisfies all the classical assumptions of the regression model, and *a*_1*i*_ represents the unobserved effect.

The model given by Equations 1, 2 is estimated via the random effect regression. This procedure is discussed in Wooldridge [(18), p. 489–490].^3^

An alternative estimation approach is the fixed-effects regression model. However, this methodology is unsuitable for our study due to its requirement that all explanatory variables must be time-varying. Since *MedianAge, PopulationSize*, and *RANK2013* are time-invariant covariates, using a fixed-effects model would result in perfect multicollinearity. This issue is demonstrated in the Table A1, which compares the estimation results of the random-effects and fixed-effects models.

Another inherent limitation of the empirical models specified in Equations 1, 2 is the variation in units of measurement among the independent variables. To address this issue, we re-estimate the following model:


(3) $$\begin{array}{l} Z{\left(\Delta ln\left(Cum\_Deaths\right)\right)}_{i\times t}\;=\;\beta_{0}+\beta_{1}Z{\left(Dum\_Vaccine\times t\right)}_{i\times t}\\ \;+\beta_{2}Z{\left(MedianAge\right)}_{i\times t}\\ \;+\beta_{3}Z{\left(PopulationSize\right)}_{i\times t}\\ \;+\beta_{4}Z{\left(RANK2013\right)}_{i\times t}+\mu_{2i,t} \end{array}$$

(4) $$\begin{array}{ccc} \mu_{2i,t}\; & = & \;a_{2i}+\in_{2,i,,t} \end{array}$$

where $Z{\left(X\right)}_{i\times t}\;=\;\frac{X_{i\times t}-\bar{X}}{S_{X}},\bar{X}$ is the mean and S_X_>0 is the standard deviation of X_*i*×*t*_.^4^ The advantage of this model lies in the equal units of measurement, where increase in one unit of measurement indicates one standard deviation. Consequently, the estimated parameters β_1_, β_2_, β_3_, β_4_ measure the separate contribution of each independent variable following an identical increase in the unit of measurement. It may be readily verified that for a normalized regression model with a constant term and one explanatory variable, the coefficient of the normalized explanatory variable yields the Pearson correlation between the two variables.^5^

This method, also referred to as the standardized or beta coefficient approach, is discussed in detail by Wooldridge [(18), p. 187–189]. It offers both advantages and disadvantages.

*Advantage*: Standardization ensures that all explanatory variables are measured on a consistent scale, facilitating easier interpretation. In particular, a one-unit change in a standardized independent variable corresponds to a one-standard-deviation change, allowing for more meaningful comparisons of effect sizes across variables.

This approach can be particularly useful—even when compared to models that apply uniform units, such as the double-log model. While the double-log model yields elasticities (i.e., the effect of a 1% increase in an independent variable, “holding” others constant), it may not provide meaningful comparisons when variables differ significantly in their range of variation. For instance, if one variable (e.g., income level in a given state) exhibits substantial variation, while another (e.g., per-student spending) shows minimal variation, comparing their respective elasticities can be misleading. In such cases, standardized (or beta) coefficients offer a more sensible basis for comparison [(18), p. 187–189].

To support our use of beta coefficients, we calculated the standard deviation-to-mean ratio. The results of this exercise is reported in Table 2 and reveals substantial differences in variability across variables. Notably, while population size shows a high degree of variation (184.94%), the variation in median age is relatively low (22.26%). This further underscores the value of standardization for meaningful comparisons across variables.

**Table 2:** The STD-mean ratio.

**Variable**	**SD**	**Mean**	**Ratio**
*Dum_vaccine*	0.4767	0.6507	73.26%
*MedianAge*	6.40	28.75	22.26%
*PopulationSize*	96,486.48	52,172.13	184.94%
*RANK*2013	74.1146	118.1978	62.70%

This ratio reflects the relative variation of the explanatory variables in percentage terms, thereby offering additional justification for reporting the standardized (beta) coefficients.

*Disadvantage*: when normalization is used, it can be more challenging to select the correct model specification, as the original scale of the variables is lost, and the model's performance might be affected by the normalization process.

We estimated the standardized regression model using the random-effect specification. This approach consists of two steps:

1. *Standardizing all variables* using the Stata command:

$$\begin{array}{c} \text{egen z\_}X_{i}\;=\;std\left(X_{i}\right) \end{array}$$

where *i* = 1, 2, 3, 4, 5 and the variables are

▘ X_1_ = Δ*ln*(Cum_Deaths )_*i, t*_
▘ X_2_ = Du*m* Vaccine*i, t*×*t*
▘ X_3_ = MedianAge*i*
▘ X_4_ = PopulationSize*i*
▘ X_5_ = RANK2013_*i*_

2. *Estimating the random-effects regression* using the standardized variables with the Stata command:

$$\begin{array}{c} \text{xtreg }{z\text{\_}X}_{1}\;{z\text{\_}X}_{2}\;z\text{\_}X_{3}\;{z_{X}}_{4}\;z\text{\_}X_{5},\text{ re} \end{array}$$

This model corresponds to Equations 3, 4 above. The time-invariant variables also exhibit a cross-sectional dimension—that is, variation across cities. This is evident in Tables 1, 2, where the standard deviations are clearly non-zero. Accordingly, we believe that the standardized beta coefficients are valid within the random-effects framework.

## 4 Results

This section presents the estimation results of the empirical models described in the previous section. Table 3 summarizes these findings. Columns (1) and (2) display the results from estimating Equations 1–4 using a random-effects approach, which accounts for serial correlation in the city-specific dummy variables over time [see, for example, (18), p. 489–490]. Column (2) provides the results from the standardized model given by Equation 2.

**Table 3:** Regression analysis and covariates weights.

**Variables**	**(1)**	**VARIABLES**		**(2)**
	Δln(Cum_Deaths)			𝒵 [Δln(*Cum_Deaths*]
Constant	−0.00223^***^	Constant		−0.00646
	(0.000392)			(0.00917)
*Dum_vaccine*×*t*	−5.25 × 10^−7***^	𝒵[*dum_vaccine × t*]	(4)	−0.0149^***^
	(1.33 × 10^−7^)			(0.00379)
*MedianAge*	0.000132^***^	𝒵[*Median*]	(2)	0.114^***^
	(1.81 × 10^−5^)			(0.0155)
*PopulationSize*	1.32 × 10^−8***^	𝒵[*PopulationSize*]	(1)	0.171^***^
	(7.87 × 10^−10^)			(0.0102)
*RANK*2013	−7.47 × 10^−6***^	𝒵[*RANK2013*]	(3)	−0.0743^***^
	(1.54 × 10^−6^)			(0.0153)
Observations	70,085	Observations		70,085
CityCode	167	CityCode		167
Wald Chi^2^(4)	409.48^***^			688.35^***^
Coef (*Dum_vaccine* × *t*)+ Coef (*MedianAge*)				0.0988^***^ [0.0574, 0.1401]
Coef (*Dum_vaccine* × *t*) + Coef (*PopulationSize*)				0.1561^***^ [0.1278, 0.1845]

ln(Cum_Deaths) is an approximation to the daily growth rate of COVID19 mortality. t is the time variable (*t* = 11, 12, 13, ⋯ , 560 where 11 = March 11, 2020 (Beginning of the COVID19 pandemic); 285 = December 20, 2020 (first date of vaccination); 560 = September 21, 2021).

Column (1) presents the results from the following random effects regression model: $y_{it}-\lambda {\bar{y}}_{i}\;=\;\beta_{0}\left(1-\lambda \right)+\beta_{1}\left(x_{it1}-\lambda {\bar{x}}_{i1}\right)+\cdots +\beta_{k}\left(x_{itk}-\lambda {\bar{x}}_{ik}\right)+\left(\nu_{it}-\lambda {\bar{\nu }}_{i}\right)$, where $\lambda \;=\;1-{\left[\frac{\sigma_{u}^{2}}{\left(\sigma_{u}^{2}+T\sigma_{a}^{2}\right)}\right]}^{\frac{1}{2}}$ and ν_*it*_ = *a*_*i*_+*u*_*it*_.

Here, *y*_*it*_ is the dependent variable, while *x*_*it*1_, ⋯ , *x*_*itk*_ are the independent variables. The coefficients β_0_, β_1_, ⋯ , β_*k*_ denote the parameters to be estimated. The term *a*_*i*_–refers to a vector of generic dummy variables (one for each locality, excluding the base category). The error term *u*_*it*_ represents the classical random disturbance, which satisfies the standard assumptions of the OLS regression model. Importantly, the composite error term ν_*it*_ exhibits serial correlation over time, which is addressed within the random effects regression framework [(18), p. 489–490].

Column (2) reports the random-effect regression analysis where each variable is converted to the standard normal distribution function: $\left(X_{i}\right)\;=\;\frac{X_{i}-\bar{X}}{\sigma_{X}}$ where $\bar{X}$ is the average and σ_X_ is the standard deviation of X_*i*_. The relative weight of each variable is given in the left side of column (2).

99% confidence intervals are given in square brackets. Standard errors are given in parentheses.

^***^p < 0.01.

The findings suggest that the projected mortality growth rate increases with both median age and population size, while it decreases with higher vaccine availability and improved municipal socio-economic rankings.

Table A1 compares the random-effects and fixed-effects estimation methods. Despite differences in model specifications, both approaches yield consistent estimates for the impact of the vaccine era—the only time-varying covariate—on mortality rates. Specifically, the fixed-effects model estimates a daily decline in mortality of approximately 5.25 × 10^−7^ during the vaccine era, while the random-effects model indicates a slightly smaller reduction of 4.62 × 10^−7^ per day.

**Figure 1:**
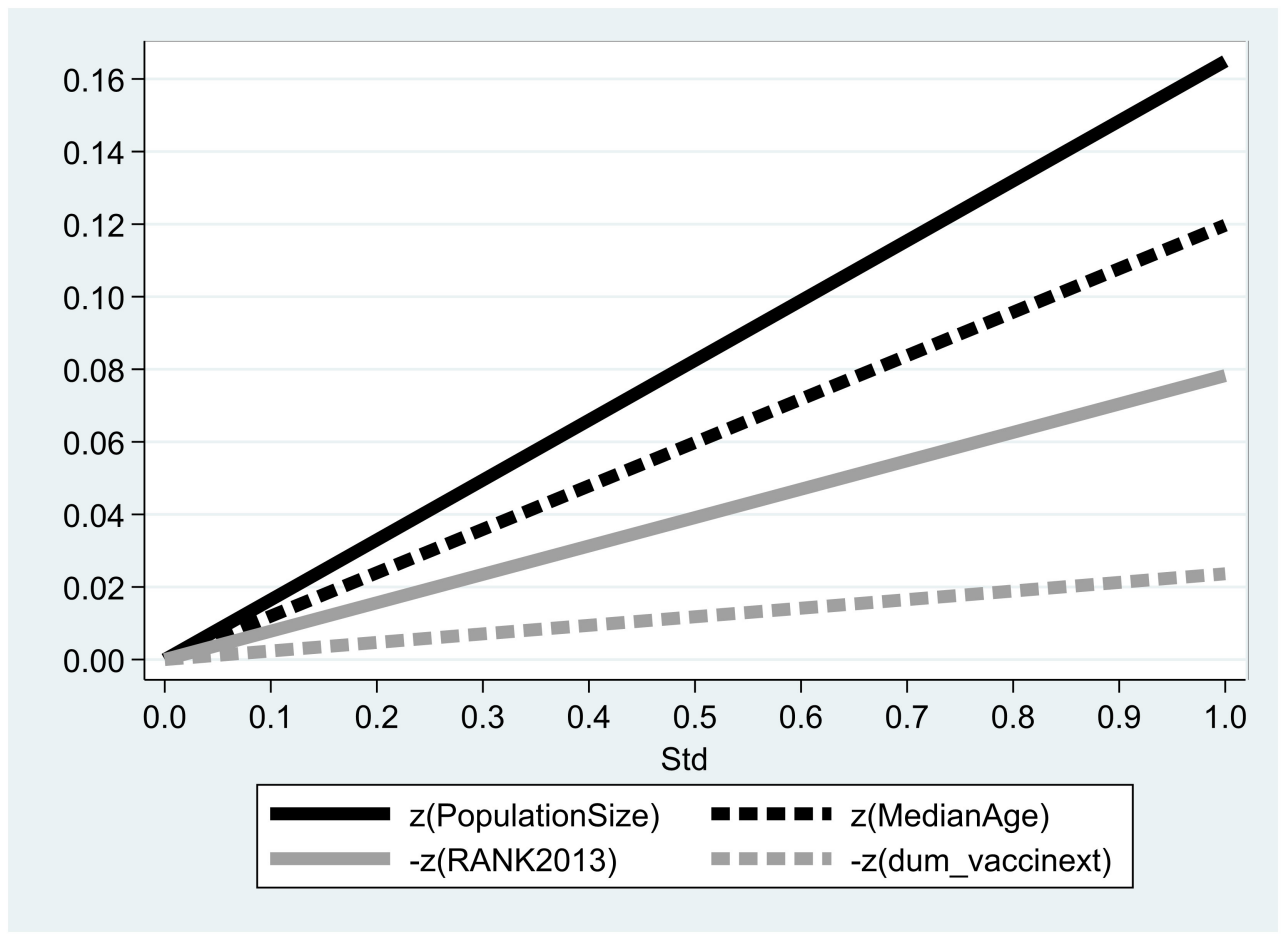
Relative contributions of explanatory variables. The figure describes the relative contribution of each variable to the daily mortality rate from COVID19 following a one standard deviation increase and is based on the outcomes obtained from column (2) in Table 3. Std equals 1 for one standard deviation from the mean of each variable.

Figure 1 illustrates the relative impact of each variable on the daily COVID-19 mortality rate following a one standard deviation increase. This figure is based on the results from Column (2) of Table 3. A standard deviation (SD) represents one standard deviation from the mean of each variable. The figure shows that the largest impacts on the mortality growth rate come from the population size and median age, with a one standard deviation increase in these variables corresponding to increases of 0.171 and 0.114 standard deviations, respectively. Conversely, the smallest impacts are observed from a one standard deviation decrease in socio-economic ranking (0.0743) and vaccination availability (0.0149).

Referring to the COVID19 vaccinations, the outcomes demonstrate that they are less effective in the case of infection, which is a necessary condition of mortality from the disease. Infected persons belong to a separate risk group. This, in turn, raises the contingent likelihood of mortality from the disease. The main contribution to mortality prospects is population size.^6^ This may be explained on the grounds of elevated number of interactions in more crowded cities (6).

The outcomes reported at the bottom of Table 3 further corroborate these findings. In the case of one standard deviation increase of both median age and availability of vaccinations, the combined contribution to mortality growth rate is still positive [+0.0988 with 99% confidence interval (0.0574, 0.1401)]. The null hypothesis according to which the coefficients of these two variables offset each other is rejected at the 1% significance level.

In the case of one standard deviation increase of both population size and availability of vaccinations, the combined contribution to mortality growth rate is still positive [+0.1561 with 99% confidence interval (0.1278, 0.1845)]. The null hypothesis according to which the coefficients of these two variables offset each other is rejected at the 1% significance level.

## 5 Discussion

This study employs panel data to analyze the COVID-19 mortality growth rate across 167 municipalities in Israel from March 2020 to September 2021. The findings indicate several key factors influencing mortality rates. Population size emerges as the primary contributor, likely due to increased interactions in densely populated areas. Additionally, the median age of the population shows a significant positive association with mortality growth rate, reflecting age's established role as a risk factor for COVID-19 severity. In contrast, socio-economic ranking and vaccination status exhibit negative associations, although higher vaccination rates and older populations still correlate with increased mortality growth rates.

The study underscores the importance of tailored public health interventions. Given reduced vaccine efficacy in already-infected individuals, early identification and targeted medical interventions are crucial. Municipal-level coordination is recommended to implement strategies such as timely medication provision and medical outreach to high-risk groups. These efforts are essential for mitigating COVID-19 mortality disparities across diverse socio-demographic settings.

Limitations of the study include its focus on data solely from 2020 to 2021, without accounting for subsequent changes in coronavirus strains. To enhance the study's relevance, future iterations should consider extending the timeframe to include 2022 and 2023 to capture evolving viral dynamics more comprehensively. Additionally, exploring the critical mass of vaccinated individuals needed to achieve herd immunity against COVID-19 would provide valuable insights for future research directions.

## 6 Conclusions

To investigate the COVID19 mortality growth rate, panel data was used in this study. As part of the study, we observe the rate of increase in mortality of COVID19 in each of the cities and localities sampled in Israel from March 11, 2020 (first documentation of COVID19 cases) to September 21, 2021 (date of availability of vaccines, December 20, 2020).

Israel provides an interesting case study of the COVID19 pandemic, with three salient features: (1) High urbanization levels and non-uniform distribution of population densities, which, in turn, might increase the spread of the pandemic (6, 7); (2) Disparities in household income groups and socio-economic ranking (8); and (3) The early initiation of a nationwide vaccination campaign, which resulted in the full vaccination (i.e., receipt of two vaccine doses) of more than half the population by the end of March 2021 (9).

The objective of the current study is to investigate the relative contribution of the following factors to the daily COVID19 mortality growth rate: vaccination status, population size, socio-economic ranking and median age of population of 167 municipalities, covering almost 94 percent of the Israeli population.

The conventional empirical model uses different units of measurement of each explanatory variable. Unlike previous studies, we estimate a model with identical units of measurement, namely one standard deviation of each independent variable. This target is achieved by normalizing all the variables in the regression model.

Findings suggest population size of cities as the factor providing the highest contribution to projected mortality growth rate increase. This may be explained on the grounds of elevated number of interactions in more crowded cities (6, 14, 15). Compared to population size, the second factor producing positive contribution to COVID19 projected death growth rate is the median age of the population in the municipality. Indeed, the academic literature to date shows that a salient risk factor for infection and mortality from SARS-COV2 is age (2, 10, 11). The components providing a negative contribution include socio-economic ranking and vaccination. The outcomes also show that a one standard deviation *rise* in both vaccination and median age is still associated with an increase in COVID19 projected mortality growth rate.

Given the reduced effectiveness of the BNT162b2 vaccine for the group of individuals who are already infected from SARS-COV2 virus, which is a necessary condition of mortality from the disease, care should be taken in an effort to accelerate the COVID19 identification. Therefore, the public policy repercussions of our study include the need for better coordination at a municipal level between health and municipal and welfare services, particularly in large cities. Examples may include: (1) Reporting on isolated individuals above 65 years or those belonging to risk factor groups to health authorities; (2) immediate intervention by provision of medication, such as Regeneron, efficiently given one-three days after the COVID19 identification (16); (3) a complementary nursing and medical intervention strategy, such as, directed phone or house calls; (4) training of municipal employees to generate a higher awareness to risk factors (e.g., age, comorbidity, vaccination status), particularly given that antibody responses were reduced for susceptible populations and therefore they might be more prone to breakthrough infections (17). (5) Supply of monitoring and saturation devices for risk factor groups.

## Data Availability

The original contributions presented in the study are included in the article/supplementary material, further inquiries can be directed to the corresponding author.

## Footnotes

^1^This implies the use of an unbalanced panel structure, as detailed in the subsequent paragraph.

^2^This principle may be readily demonstrated as follows. An increase from 10 to 11 deaths per day reflects an exact growth of 10%:

$$\begin{array}{c} \frac{11}{10}-1\;=\;0.1\;=\;10\% \end{array}$$

The corresponding log difference is:

$$\begin{array}{c} ln11-ln10\;=\;0.0953\;=\;9.53\%\approx 10\% \end{array}$$

This illustrates that the log difference closely approximates the actual percentage change.

^3^According to Wooldridge, the random effect regression model is given by:

$$\begin{array}{c} y_{it}-\lambda {\bar{y}}_{i}\;=\;\beta_{0}\left(1-\lambda \right)+\beta_{1}\left(x_{it1}-\lambda {\bar{x}}_{i1}\right)+\cdots +\beta_{k}\left(x_{itk}-\lambda {\bar{x}}_{ik}\right)+\left(\nu_{it}-\lambda {\bar{\nu }}_{i}\right) \end{array}$$

where

$$\begin{array}{l} \;\lambda \;=\;1-\sigma_{u}^{2}/{\left(\sigma_{u}^{2}+T\sigma_{a}^{2}\right)}^{\frac{1}{2}}\;\\ \nu_{it}\;=\;a_{i}+\;u_{it}\$. \end{array}$$

Here, *y*_*it*_ is the dependent variable, while *x*_*it*1_, ⋯ , *x*_*itk*_ are the independent variables. The coefficients β_0_, β_1_, ⋯ , β_*k*_ denote the parameters to be estimated. The term *a*_*i*_–refers to a vector of generic dummy variables (one for each locality, excluding the base category). The error term *u*_*it*_ represents the classical random disturbance, which satisfies the standard assumptions of the OLS regression model. Importantly, the composite error term ν_*it*_ exhibits serial correlation over time, which is addressed within the random effects regression framework [(18), p. 489–490].

^4^When X_*i*×*t*_ is constant, S_X_ = 0, making the transformation mathematically ill-defined. Including β_0_ means estimating the model without standardizing the intercept. However, under these conditions, one should expect to fail to reject the null hypothesis that the intercept is zero. This is because, when the dependent variable is standardized, its sample mean becomes zero, and the intercept in the regression reflects this mean.

^5^This may be demonstrated as follows. The simple regression model with one explanatory variable is defined as: (1) $Y_{i}\;=\;\hat{\alpha }+\hat{\beta }X_{i}+{\hat{\mu }}_{i}$ where Y_*i*_ is the dependent variable, X_*i*_ is the independent variable, ($\bar{X},\bar{Y}$) are the sample means, (S_X_, S_Y_) are the respective standard deviations, $\hat{\alpha },\hat{\beta }$ are the estimated parameters via the OLS procedure, ${\hat{\mu }}_{i}$ are the residuals, ${\hat{\beta }}_{OLS}\;=\;\frac{COV\left(X_{i},Y_{i}\right)}{S_{X}^{2}}$ and the Pearson correlation is: $r_{X,Y}\;=\;\frac{COV\left(X_{i},Y_{i}\right)}{S_{X}S_{Y}}\;=\;\hat{\beta }\frac{S_{X}}{S_{Y}}$ [e.g., (13), p. 237–243]. The derived normal equations of the OLS procedure yield: (2) $\bar{Y}\;=\;\alpha +\beta \bar{X}$. Subtracting (2) from (1) and rearranging terms yields: (3) $\left(Y_{i}-\bar{Y}\right)\;=\;\hat{\beta }\left(X_{i}-\bar{X}\right)+{\hat{\mu }}_{i}$. Multiplication of (3) by $\frac{1}{S_{X}S_{Y}}$ yields: (4) $\frac{\left(Y_{i}-\bar{Y}\right)}{{S_{X}S}_{Y}}\;=\;\hat{\beta }\frac{\left(X_{i}-\bar{X}\right)}{{S_{X}S}_{Y}}+{\hat{\mu }}_{i}$. Substitution of $Z_{Y}\;=\;\frac{\left(Y_{i}-\bar{Y}\right)}{S_{Y}}$ and $Z_{X}\;=\;\frac{\left(X_{i}-\bar{X}\right)}{S_{X}}$ yields: (5) $\frac{Z_{Y}}{S_{X}}\;=\;\hat{\beta }\frac{Z_{X}}{S_{Y}}+{\hat{\mu }}_{i}$. Multiplication by S_X_ yields: (6) $Z_{Y}\;=\;\left[\hat{\beta }\frac{S_{X}}{S_{Y}}\right]Z_{X}+{\hat{\mu }}_{i}$. Substitution of $r_{X,Y}\;=\;\hat{\beta }\frac{S_{X}}{S_{Y}}$: in Equation 6 yields: (7) $Z_{Y}\;=\;r_{X,Y}Z_{X}+\;{\hat{\mu }}_{i}$.

^6^City density is not always correlated with city size. The Pearson correlation between these two variables is 0.3814.

## Appendix

**Table A1:** Fixed vs. random effect regression.

**Procedure**	**(1)**	**(2)**
	**Random effect**	**Fixed-effect**
**Variables**	Δln(Cum_Deaths)	Δln(Cum_Deaths)
Constant	–0.00223^***^	0.00141^***^
	(0.000392)	(4.60 × 10^–5^)
*Dum_vaccine*×*t*	–5.25 × 10^–7^^***^	–4.62 × 10^–7^^***^
	(1.33 × 10^–7^)	(1.34 × 10^–7^)
MedianAge	0.000132^***^	–
	(1.81 × 10^–5^)	–
PopulationSize	1.32 × 10^–8^^***^	–
	(7.87 × 10^–10^)	–
RANK2013	–7.47 × 10^–6^^***^	–
	(1.54 × 10^–6^)	–
Observations	70,085	70,085
Number of CityCode	167	167
Wald Chi2(4)	409.48^***^	–
*F*_(1, 69, 917)_	_−−_	11.90^***^

Standard errors are given in parentheses.

*** P < 0.01.
